# Two New Compounds Isolated from *Liriope muscari*

**DOI:** 10.3390/molecules17088773

**Published:** 2012-07-25

**Authors:** Wen-Jie Li, Zhi-Hao Zhang, Xian-Long Cheng, Jing Liu, Yi He, Chao Zhou, Ying Guo, Rui-Chao Lin, Gang-Li Wang

**Affiliations:** 1 National Institutes for Food and Drug Control, Beijing 100050, China; E-Mails: lwj115@163.com (W.-J.L.); zzh-198518@163.com (Z.-H.Z.); 2 Faculty of Chinese Medicine, Macau University of Science and Technology, Macau, China; 3 QiQiHaEr Institute for Food and Drug Control, QiQiHaEr 161000, China

**Keywords:** liliaceae, *Liriope muscari*, norcurlignan, limlactone, antioxidant activity

## Abstract

Two new compounds, (2*S*,3*R*)-methyl 7-hydroxy-2-(4-hydroxy-3-methoxy-phenyl)-3-(hydroxymethyl)-2,3-dihydrobenzofuran-5-carboxylate (**1**) and (4*R*,5*S*)-5-(3-hydroxy-2,6-dimethylphenyl)-4-isopropyldihydrofuran-2-one (**2**), tentatively named norcurlignan and limlactone, respectively, were isolated from *Liriope muscari*, together with the known compound (−)-pinoresinol (**3**). The structures of these compounds were elucidated and characterized on the basis of 1D NMR, 2D NMR, CD and MS data. The *in vitro* antioxidant activities of compounds **1**–**3** were assessed by the DPPH and ABTS scavenging methods.

## 1. Introduction

*Liriope muscari* (Decne.) Bailey (Liliaceae) is called *duantingshanmaidong* in China. Due to the lack of Radix Ophiopogonis (*maidong* in Chinese) resources and the similar pharmacological activities of these two herbal medicines, *L. muscari* is used locally in *Fujian* province as a substitute for *maidong* [1]. *Maidong* is a traditional herbal medicine widely used in China as a tonic agent. Pharmacology studies also showed that this herbal medicine has a positive effect on various inflammation-related diseases [2], however, its antioxidant activity was seldom reported [3].

Previously, in the chemical study of genus *Liriope* (Liliaceae), it was proven that the main constituents in the roots of the genus were polysaccharides and steroidal glycosides [4,5,6,7]. Other kinds of constituents were seldom reported. However, with the deepening of the research on *Liriope*, other kinds of compounds were isolated, including eudesmane sesquiterpene [8,9,10], benzofuran derivatives [11], and phenolic compounds. In our previous study [12], five phenolic compounds were isolated from *L. muscari* and their antioxidant activities were reported. In continuation of our chemical studies of *L. muscari*, we describe herein the isolation and structural elucidation of three additional compounds, including two new ones—(2*S*,3*R*)-methyl 7-hydroxy-2-(4-hydroxy-3-methoxyphenyl)-3-(hydroxymethyl)-2,3-dihydrobenzofuran-5-carboxylate (**1**) and (4*R*,5*S*)-5-(3-hydroxy-2,6-dimethylphenyl)-4-isopropyldihydrofuran-2-one (**2**)—which were tentatively named norcurlignan and limlactone, respectively, and the known compound (−)-pinoresinol (**3**), which was isolated for the first time from the genus *Liriope*. Since these compounds contain phenolic hydroxyl groups indicating potential antioxidant activity, experiments were also carried out to evaluate their antioxidant activities.

## 2. Results and Discussion

### 2.1. Structure Analysis and Characterization of Compounds ***1**–**3***

The compounds were isolated using silica gel and Sephadex LH-20 gel column chromatography from an 80% ethanol extract of *L. muscari.* The structures of compounds **1**–**3** were characterized by examination of their HR ESI-MS, NMR (1D and 2D) data, CD spectra and comparison with literature reports.

Compound **1** was obtained as a colorless solid; ${\left[\alpha \right]}_{D}^{25}$ +52.3° (*c* 0.016, CH_3_OH). UV (CH_3_OH) λ_max_ (nm) (lgε): 274.4 (4.3). Its molecular formula was assigned as C_18_H_18_O_7_, suggesting ten degrees of unsaturation, on the basis of the [M−H]^−^ ion peak at *m*/*z* 345.0962 (calcd. for C_18_H_17_O_7_, 345.0974) in the HR-ESI-MS. ^1^H-NMR (DMSO-*d*_6_, 600 MHz) showed eight downfield proton signals, including two phenolic hydroxyl group signals (1H, δ 9.70; 1H, δ 9.10), an AB-pattern for two aromatic protons at δ 7.40 (1H, d, 1.8 Hz) and δ 7.33 (1H, d, 1.8 Hz), and an ABX-pattern for three aromatic protons at δ 6.93 (1H, d, 1.8 Hz), δ 6.78 (1H, dd, 7.8 Hz, 1.8 Hz), and δ 6.75 (1H, d, 7.8 Hz). Ten highfield proton signals, including two singlets (3H, δ 3.77, OCH_3_; 3H, δ 3.74, OCH_3_), a doublet (1H, δ 5.52) coupled to a multiplet (1H, δ 3.51) and a double doublet (2H, δ 3.68) were also observed. The ^13^C-NMR data (Table 1) of **1** was similar to that of curlignan [10], the major difference being that the methoxy group at C-7 was replaced by a hydroxyl group on the basis of the chemical shift for the C-7 carbon (δ 141.4).

**Figure 1:**
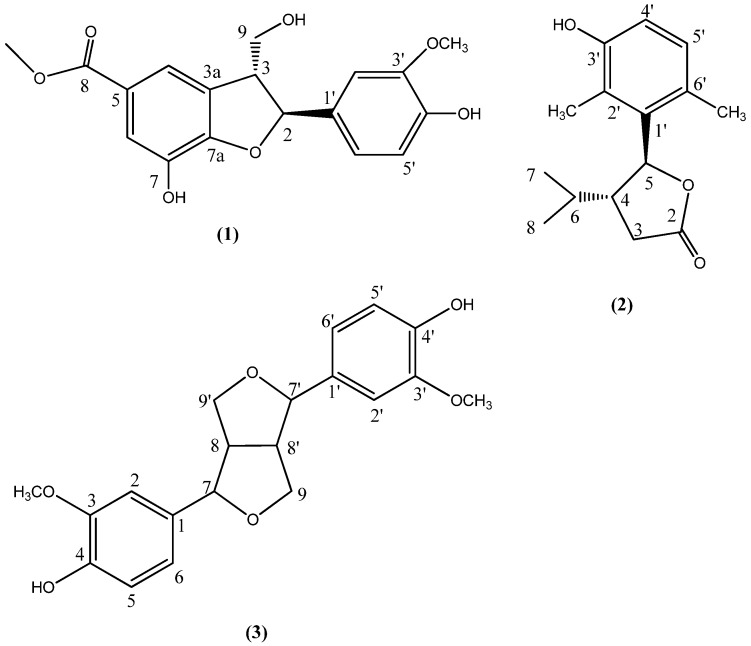
Structures of compounds isolated from *L. muscari*.

**Figure 2:**
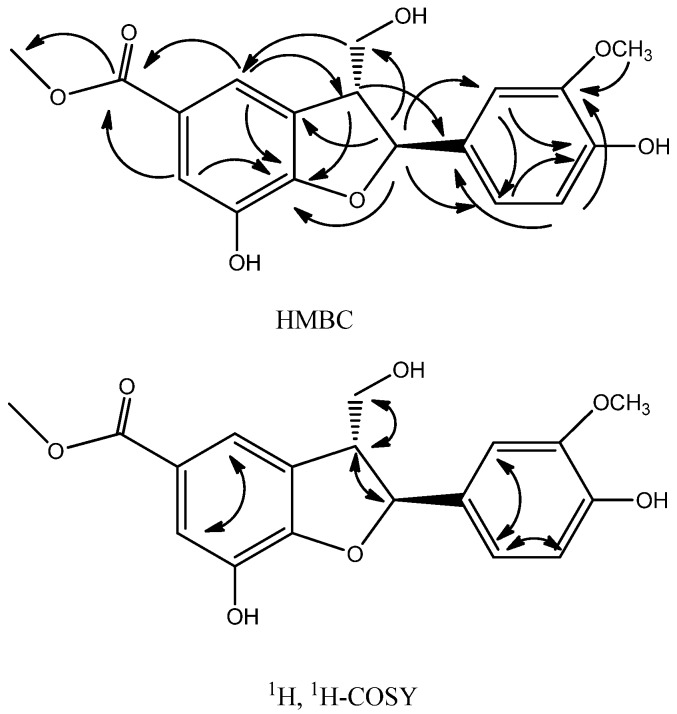
Key HMBC and ^1^H, ^1^H-COSY correlations of compound **1**.

These data together would suggest compound **1** to be a 5-(3-hydroxy-2,6-dimethylphenyl)-4-isopropyldihydrofuran-2-one (Figure 1). The assigned structure for compound **1** was confirmed by analysis of the HMBC spectrum and ^1^H-^1^H COSY (Figure 2).

The absolute stereochemistry at C-2 and C-3 of compound **1** was determined by NOE experiments and its CD spectrum. In the NOE spectrum, irradiation at H-2 signal caused the enhancement of the H-9 signal while H-3 signal was not enhanced, which indicated that H-2 and H-3 were *trans*-oriented. The absolute stereochemistry at C-2 and C-3 of compound **1** was determined to be 2*S*,3*R* by comparison of its CD curve with that of curlignan [13], both showing one negative and one positive Cotton effect (CE) around 230 and 280 nm, respectively. These data established the structure of compound **1** as (2*S*,3*R*)-methyl 7-hydroxy-2-(4-hydroxy-3-methoxyphenyl)-3-(hydroxymethyl)-2,3-dihydrobenzofuran-5-carboxylate. This novel natural product is tentatively named norcurlignan after its homologue curlignan.

Compound **2** was obtained as colorless feathery crystals; ${\left[\alpha \right]}_{D}^{25}$ −2.0° (*c* 0.0075, CH_2_Cl_2_), UV (CH_3_OH) λ_max_ (nm) (lgε): 289.5 (3.2). Its molecular formula was assigned as C_15_H_20_O_3_, suggesting six degrees of unsaturation, on the basis of the [M−H]^−^ ion peak at *m*/*z* 247.1331 (calcd. for C_15_H_19_O_3_, 247.1334) in the HR-ESI-MS. ^1^H-NMR (CDCl_3_, 500 MHz, Table 2) revealed an AB-pattern for the two aromatic protons at δ 6.88 (1H, d, 8.5 Hz) and δ 6.68 (1H, d, 8.5 Hz), indicating the presence of a tetrasubstituted benzene ring. 

Sixteen ^1^H signals appeared at highfield, including a doublet at δ 5.62 (1H, d, 9.0 Hz ); a multiplet (1H, δ 3.51); a methylene signal (1H, δ 2.70; 1H, δ 2.52); two singlets (3H, δ 2.32; 3H, δ 2.26) and a group of isopropyl proton signals (1H, δ 1.75; 3H, δ 0.98; 3H, δ 0.79). The ^13^C-NMR data (Table 2), DEPT and HSQC spectra of compound **2** allowed the assignment of 15 carbon signals to one secondary, five tertiary, five quaternary carbons, and four methyls, further suggesting **2** be a 4-phenyldihydrofuran-2-one.

**Figure 3:**
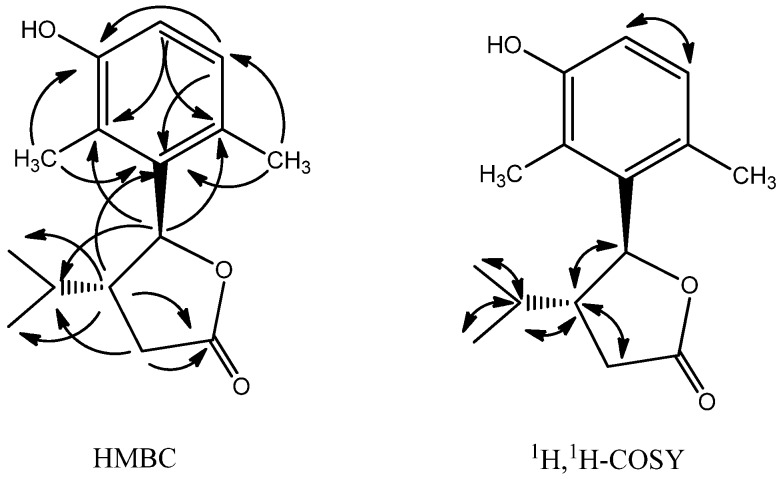
Key HMBC and ^1^H, ^1^H-COSY correlations of compound **2**.

In the HMBC spectrum (Figure 3), the observation of diagnostic correlations from H-5 to C-2′ and C-6′, allowed the tetrasubstituted phenyl group to be attached to C-5. The isopropyl group was assigned to C-4, based on the correlations of H-7 and H-8 with C-4, and the correlations of H-3 and H-5 with C-6. The ester carbonyl group was assigned to C-2, based on the correlations of H-4 and H-3 with C-2. The positions of the phenyl group substituents were established by the HMBC spectrum. These data pooled together would suggest compound **2** to be a 5-(3-hydroxy-2,6-dimethylphenyl)-4-isopropyldihydrofuran-2-one.

The assigned structure for compound **2** was confirmed by the analysis of the ^1^H-^1^H COSY spectrum, which further revealed the presence of a CH (H-5)-CH (H-4)-CH_2_ (H-3), CH_3_-CH (H-6)-CH_3_ fragment (Figure 3).

**Figure 4:**
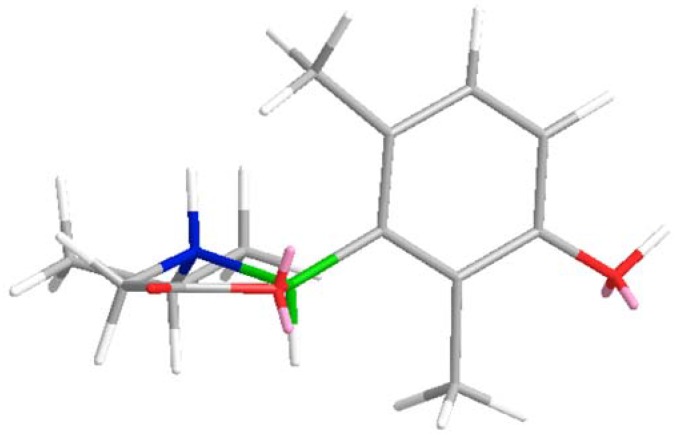
3D-stucture model of compound **2** in minimum energy state.

The absolute stereochemistry at C-4 and C-5 was determined on the basis of the coupling constants and the CD spectrum. According to the coupling constant between H-4 and H-5 (*J* = 9.0 Hz), H-4 and H-5 was determined to be *trans*-oriented [14], so there were two possible configurations which were (4*R*,5*S*) or (4*S*,5*R*). According to literature on the CD spectra of *γ*-lactone rings [15], the C-CO-O-C group tends to form a coplanar conformation. When observed from a specific location, if *β*-C (C-4) is above the plane, the CE associated with the lactone n→π* transition should be positive, as it is observed in the CD spectrum at 224 nm. Through Chembio 3D simulation, it was found that only if the configuration is (4*R*,5*S*) (Figure 4), is it possible that *β*-C (C-4) is above the plane in the minimum energy state. In summary, the absolute configuration was determined to be (4*R*,5*S*). These data established the structure of compound **2** as (4*R*,5*S*)-5-(3-hydroxy-2,6-dimethylphenyl)-4-isopropyl-dihydrofuran-2-one. This novel natural product is tentatively named limlactone.

### 2.2. *In Vitro* Antioxidant Activity

DPPH and ABTS radical scavenging assays were carried out to evaluate the antioxidant activities of compounds **1**–**3** using vitamin C (VC) and butylated hydroxytoluene (BHT) as positive controls. The results are shown in Table 3. In both methods, compounds **3** showed potential activity, which was consistent with the reported results [16]. Compounds **1** and **2** showed potential activity in the ABTS assay. The DPPH and ABTS assay were carried out using the same methods reported in the previous paper [12].

## 3. Experimental 

### 3.1. General

^1^H-and ^13^C-NMR spectra were recorded on Bruker Avance DRX 500 instrument or Varian Unity VNS 600 using DMSO-*d*_6_ or CDCl_3_ as solvent, with TMS as internal standard. Agilent 6320 Ion TRAP LC/MS and Waters Xevo^TM^ UPLC-QTof were employed for MS analysis. The specific rotation was recorded on AUTOPOL IV Automatic Polarimeter (Rudolph, Hackettstown, NJ, USA). UV spectra were recorded on an Agilent 8453 UV/Vis Spectrophotometer (Agilent, Santa Clara, CA, USA). CD spectra were taken on a JASCO J-815 Spectropolarimeter (JASCO, Tokyo, Japan) using a 0.1 cm standard cell and spectrophotometric-grade MeOH. IR spectra were taken on a Nicolet 5700 FTIR Spectrometer (Thermo, Waltham, MA, USA). In the antioxidant assay, a SpectraMax 190 Absorbance Microplate Reader (Molecular Devices, Sunnyvale, CA, USA) and 96 Well Cell Culture Cluster (Costar, Corning, NY, USA) were used. 1,1-Diphenyl-2-picrylhydrazyl (DPPH) and 2,2′-azino-bis (3-ethylbenzthiazoline-6-sulfonic acid) (ABTS) were purchased from Sigma (Sigma-Aldrich GmbH, Steinheim, Germany). Sephadex LH-20 was purchased from Amersham Pharmacia Biotech AB (Uppsala, Sweden). Polyamide resin (100–200 mesh) was purchased from Beijing Zhongxiyuanda Technical Co. Ltd. (Beijing, China). Silica gel (160–200 mesh, 200–300 mesh) for column chromatography was purchased from Qingdao Marine Chemical Plant (Qingdao, Shandong Province, China). All other chemicals were of analytical reagent grade and used without any further purification.

### 3.2. Plant Material

Fresh fibrous roots of *L. muscari.* were collected from Quanzhou City, Fujian Province, China, in May 2010. The species was identified by Professor Zhang J. (National Institutes for Food and Drug Control, NIFDC for short). The voucher specimens were deposited at the herbarium of NIFDC. The roots were air-dried and ground to a powder using a grinding mill (Retsch Muhle, Haan, Germany).

### 3.3. Compound Isolation

The powder (2 kg) was extracted three times with hot 80% ethanol (1 L), for 1 h each time. The extracts were concentrated to afford a syrup (1 kg), which was dissolved in 10% ethanol (4 L). Polyamide (1 kg) was added into the solution which was stirred for about 1 h to make sure the phenolic compounds were adsorbed on the resin to some extent. Then the polyamide was centrifuged to dryness (1,000 ×g, 10 min). Fresh water was used to rinse the polyamide several times till the water was nearly colorless. Then 95% ethanol was used to rinse the polyamide and the solution was collected. The ethanol solution was evaporated to dryness under reduced pressure to afford a solid residue (30 g). The solid residue was chromatographed over a silica gel (160–200 mesh) column (45 × 6.0 cm i.d.) with CHCl_3_/MeOH (20:1 to 8:1) to afford 30 fractions (F01–F30). Fraction F03 (2.2 g) was subjected to Sephadex LH-20 column chromatography (120 × 2.5 cm i.d.) with CHCl_3_/MeOH (10:1) to afford 11 subfractions (F0301–F0311). Then fraction F0306 (50 mg) was chromatographed over a silica gel column (200–300 mesh, 30 × 2.0 cm i.d.) with petroleum ether/EtOAc (PE/E, 8:1 to 5:1) to afford compound **2** (6 mg). Fraction F0307 (63 mg) was chromatographed over a silica gel column (200–300 mesh, 30 × 2.0 cm i.d.) with PE/E (6:1 to 3:1) to afford compound **3** (9 mg). Fraction F06-F08 (0.8 g) was subjected to Sephadex LH-20 column chromatography (120 × 2.5 cm i.d.) with MeOH to afford fractions F0601-F0615. Fraction F0609 (100 mg) was chromatographed over a silica gel column (200–300 mesh, 30 × 2.0 cm i.d.) with PE/E (3:2 to 1:1) to afford compound **1** (23 mg).

*Norcurlignan* (**1**): Colorless solid. ${\left[\alpha \right]}_{D}^{25}$ +52.3° (*c* 0.016, CH_3_OH). UV (CH_3_OH) λ_max_ (nm) (lgε): 274.4 (4.3). HR-ESI-MS: *m*/*z* 345.0962 [M−H]^−^ (calcd. for C_18_H_17_O_7_, 345.0974). The ^1^H- and ^13^C-NMR spectral data are listed in Table 1.

*Limlactone* (**2**): Colorless feathery crystals (CHCl_3_). ${\left[\alpha \right]}_{D}^{25}$ −2.0° (*c* 0.0075, CH_2_Cl_2_). UV (CH_3_OH) λ_max_ (nm) (lgε): 289.5 (3.2). HR-ESI-MS: *m*/*z* 247.1331 [M−H]^−^ (calcd. for C_15_H_19_O_3_, 247.1334). The ^1^H- and ^13^C-NMR spectral data are listed in Table 2.

*(−)-Pinoresinol* (**3**): Colorless oil. ${\left[\alpha \right]}_{D}^{25}$ −63.1° (*c* 0.012, CH_2_Cl_2_). UV (CH_3_OH) λ_max_ (nm) (lgε): 231, 281 (4.3, 3.9). C_20_H_22_O_6_ (ESI-MS, *m*/*z* 357 [M−H]^−^). ^1^H-NMR (CDCl_3_, 500 MHz) δ: 6.92, 6.90 (4H, m, H-2, 2′, 5, 5′), 6.84 (2H, d, 8.5 Hz, H-6, 6′), 4.76 (2H, d, 3.0 Hz, H-7, 7′), 4.27 (2H, m, H-9b, 9′b), 3.90 (2H, m, H-9a, 9′a), 3.92 (6H, s, 2×OCH_3_), 3.13 (2H, m, H-8, 8′). ^13^C-NMR (DMSO-*d*_6_, 125 MHz) δ: 146.7 (C-4, 4′), 145.3 (C-3, 3′), 132.9 (C-1, 1′), 119.0 (C-6, 6′), 114.3 (C-5, 5′), 108.6 (C-2, 2′), 85.9 (C-7, 7′), 71.7 (C-9, 9′), 56.0 (2×OCH_3_), 54.2 (C-8, 8′). The ^1^H- and ^13^C-NMR spectral data are consistent with the published data [17,18].

## 4. Conclusions 

Two new compounds, norcurlignan (**1**) and limlactone (**2**), together with the known compound (−)-pinoresinol (**3**) were isolated from *L. muscari*. Their antioxidant activities were evaluated using DPPH and ABTS assays. In both methods, compound **3** showed potential activity (IC_50_ 111.8 µM, 18.5 µM). Compound **1** and compound **2** showed potential activity in the ABTS assay, with IC_50_ values of 46.4 µM and 23.1 µM, respectively.

## Figures and Tables

Blue: C-4 *R*; Green: C-5 *S*.

**Table 1:** NMR data of compound **1** (DMSO-*d*_6_, 600 MHz, 125 MHz).

Position	δ_C_	δ_H_
2	88.2	5.52 (1H, d, 6.6 Hz)
3	53.0	3.51 (1H, q, 6.6, 6.0 Hz)
3a	130.5	
4	117.9	7.40 (1H, d, 1.8 Hz)
5	122.8	
6	117.6	7.33 (1H, d, 1.8 Hz)
7	141.4	
7a	151.7	
8	166.6	
9	63.1	3.68 (2H, dd, 6.0, 1.8 Hz)
1′	132.2	
2′	110.9	6.93 (1H, d, 1.8 Hz)
3′	148.1	
4′	147.0	
5′	115.8	6.75 (1H, d, 7.8 Hz)
6′	119.3	6.78 (1H, dd, 7.8, 1.8 Hz)
8-OCH_3_	52.2	3.77 (3H, s)
3′-OCH_3_	56.1	3.74 (3H, s)

**Table 2:** NMR data of compound **2** (CDCl_3_, 500 MHz, 125 MHz).

Position	δ_C_	δ_H_
2	176.5	
3	32.1	2.70 (1H, m), 2.52 (1H, m)
4	47.8	2.75 (1H, m)
5	82.5	5.62 (1H, d, 9.0 Hz)
6	29.3	1.75 (1H, m)
7	18.6	0.98 (3H, d, 7.0 Hz)
8	21.6	0.79 (3H, d, 6.5 Hz)
1′	135.2	
2′	123.4	
3′	152.6	
4′	115.2	6.68 (1H, d, 8.5 Hz)
5′	129.7	6.88 (1H, d, 8.5 Hz)
6′	129	
2′-CH_3_	12.4	2.26 (3H, s)
6′-CH_3_	20.5	2.32 (3H, s)

**Table 3:** IC_50_ values of the antioxidant activities of compounds **1**–**3**.

Compound	IC_50-DPPH_ (µM)	IC_50-ABTS_ (µM)
Compound **1**	111.8 ± 9.0	18.5 ± 1.8
Compound **2**	--	46.4 ± 3.7
Compound **3**	43.8 ± 3.7	23.1 ± 1.5
VC	17.3 ± 1.3	52.9 ± 4.2
BHT	188 ± 15.2	25 ± 2.4

-- means IC_50_ > 200 µM; Results are means ± SD of three duplicate measurements.
